# The Position of British Nursing

**Published:** 1917-07-07

**Authors:** Henry Burdett


### THE POSITION OF BRITISH NURSING.

### By Sir Henry Burdett, K.C.B., K.C.V.O.

Sir Thomas Dewey, Ladies and Gentlemen,—I am very sorry that Sir Everard Hambro was too ill to be here to-day. He is such a constant and regular attendant, and has given up his life to the welfare of the Royal National Pension Fund, that his absence intensifies the feeling of his value to it. I am sure he has your sympathy as he has mine. I have great pleasure in seconding the adoption of the report, and I am grateful, as this honour has been conferred upon me suddenly. that Sir Thomas Dewey should be in the chair and that I should have had the privilege of listening to what he has said, because he has covered the whole ground so far as the report is concerned, and therefore I need not go into matters which he has already fully dealt with.

The Nurses' Position To-day.

What I propose to talk to you about to-day is the position of nurses. The position of nurses has gone on progressing and developing, and is now in a state of extension and growth which those of us who were instrumental in founding this Fund did not contemplate or apprehend would be possible. I think the briefest way of enforcing that view and proving it will be to remind you that the service rendered by nurses in this war has attracted the attention and won the admiration of all classes, and that there never was a time in the whole history of nursing and even of sickness when the position of a nurse was regarded as so honourable, when the responsibility attaching to it was greater, or when the honour and the nobility of the nursing profession was ever so fully realised as it is to-day. One result of all this noble work—this immense and continuous personal service—has been to have a signal recognition of it by the King in the creation of two new Orders for Women—that of the Order of the British Empire and a second that of the Companion of Honour. Now I may remind you that in the growth and development of the nursing profession and in the building up of its reputation and increasing the higher ideals of nursing, probably no single event has had more influence for good than the founding and success of the Royal National Pension Fund for Nurses. That Fund to-day has demonstrated what women can do if they have the opportunity of being thrifty, and a point I always like to bring out is that this Pension Fund is the greatest thrift agency among women workers that the world has yet seen. You can quite understand that a fact like that makes people have a very strong feeling of desire to do anything they can in the form of personal service or in more material ways to help the nurse, whoever she may be, and wherever she may be, in all the circumstances of her professional life. The position of the trained British nurse has been greatly strengthened, too, by the war and the heroism it has called forth.

### The Nurse and Her Training School.

At the present time unfortunately a cry is raised by irresponsible individuals, who are few in number but exceedingly vocal, who contend that the nurse must divorce herself from her training school, must throw over the managers of the school, especially if they are laymen, and stand out alone to enable her to seek her own salvation by becoming a politician. I do not know how that prospect may appear to the nurses who are present, but it seems to me that it would be very difficult for ambition, thirsting for individual control over a great body of women workers like our British nurses, to carry its authors much quicker to deserved defeat. Nurses have nothing to do in their profession with politics. If they are qualified they will have the right to vote, and I have no doubt they will use it wisely and well and not mix themselves up in politics. We are all looking forward to the day when the representatives of our Allies and ourselves will celebrate the advent of the great peace. Then all the Allied nations' representatives who have combined are to co-operate together to secure freedom for all workers, with the establishment and enforcement of the principles which Christ came to support and died to uphold. Is this the time for nurses to desert their profession and become active politicians? Is it not the time when they should unite as one woman to insist that there shall be unity in their ranks, for unity is strength, and it is for them, with their knowledge of the people with whom they work, their schools where they were trained, and their matrons under whom they have served, to say whom they will trust? With British nurses rests the great responsibility of providing that by co-operation and unity they enforce these views. You cannot have nurses without training schools and hospitals, and any one of you, who is old enough and has taken enough interest in the progress of nursing must realise that, it would not have been possible for this country to have had the number of trained nurses it has produced and sent to the war, if it had not been for (*a*) the existence of the Nurse-Training Schools and the Hospitals, and (*b*) the wonderful amount of financial aid which Hospital Managers, mostly laymen, have had the courage to become responsible for in order to enable British nurses to be trained in sufficient numbers.

### Our Alma Mater.

Another thing that life teaches us is that no one of high principle and character will divorce herself from the centres and associations where her earliest years were spent. We want to foster such associations, to keep them in mind, to go back to them, and to gain renewed strength from the dear memories of the past. All these considerations, nurses will agree, attach to Nurse-Training Schools and the Hospitals. I venture to say and to think, that nurses who are wise will keep free from those people who are at present sowing sedition, and talking what they call democracy, but without knowledge. For the prescription which the agitators regard as democracy reveals to us not democracy at all, but simply a scheme for the strengthening of a few small societies, which have been organised by about six individuals, that for practical purposes are entirely in the hands of their promoters, and can therefore only represent the views of a small prejudiced party, and not those of the whole body of nurses, or even of a considerable body of matrons, sisters, and trained nurses in Great Britain. British Nurses, you yourselves must unite, you must put on your thinking caps, and must decide to rally, to stand together in your own defence, and to act.

### The Layman's Life Work: An Historical Tribute.

I have something to tell you which I think will point in the direction where wisdom should lead you. Yesterday, as a Governor of St. Thomas's Hospital, I attended a Special Court of Governors, and after the meeting Mr. Wainwright, the retiring treasurer, handed me a paper. He handed it to me as the layman who had done the work of treasurer for 27 years, and who, on laying down his treasurership, was received with unanimous sympathy and an exhibition of feeling rare in public life. He is one of the hospital laymen whom a certain party are so fond of attacking. He has been the Administrative Head of the nurses and of the Nightingale School for these 27 years. Has he proved to be a tyrant, an impediment to progress, an oppressor of the nurses and nursing? Let the nurses give their spontaneous experience on these points. As he was going to the Court to lay down the treasurership he had a paper placed in his hands, and this paper is one which was prepared without the knowledge of himself or of the hospital authorities, and is, therefore, the spontaneous expression of the opinion of the Nightingale Sisters and the hundreds of nurses connected with St. Thomas's Hospital. It is signed by all the sisters, and is as follows:

**"Dear Treasurer,—**

**"The Matron has told us of your resignation as our treasurer. We, your sisters, are anxious that you should know of our sorrow and our sympathy for you this day, knowing how deeply you will feel the giving up of the lifelong work which has been your happiness and, may we say, our happiness too? May we wish you all good for the future, and offer our most grateful thanks for all you have done for our welfare and happiness all these years?—Yours sincerely and gratefully."**

Then follows the signature of every one of the forty sisters of St. Thomas's Hospital. (Cheers.) That is the answer to these sowers of disorder—the small but very active and vocal party of Divide and Disunion. They do not advance the truth. Nurses love their Alma Mater. They respect the Managers and Matrons of their Hospitals and Training Schools, who, in the majority of cases, are among the best friends women in nursing ever have. As far as a political campaign among nurses is concerned, I am glad to think it is pre-doomed to failure at its birth. You will see from this instance of Mr. Wainwright, as I know from visiting all these institutions, that the actual feeling between the Managers, Matrons and their staffs and the nurses who are trained under them in this country is cordial, sound and appreciative. The business of nursing to-day has been greatly improved, as I think you will agree, and the resulting harmony and satisfaction are manifest.

### The College of Nursing.

Yesterday Mr. Wainwright retired from the treasurership of St. Thomas's Hospital and his place was taken by the Hon. Arthur Stanley, C.B., M.P., the President of the Joint War Committee of the Red Cross, who is also the Chairman of the College of Nursing. Mr. Stanley has shown how deep is his interest in nurses and how much he will sacrifice to secure unity and to do everything possible to help them. I am quite confident from what I know that so soon as the Privy Council hand over the Royal Charter to the College—which is now due to take place, the details having been practically arranged, I understand—you will find that the College will go forward, and go forward quickly indeed. Now, it is not to their credit that a number of nurses are waiting to see which way the cat jumps. That is not a position for a trained nurse and intelligent woman to take at all. It is the interest of herself and her profession that she has to consider, and I am grateful to know that 2,000 fully-trained nurses have since January spontaneously come forward through *The Nursing Mirror* and entered their names in the Register of the College. I have worked with and for nurses for nearly half a century, and I am confident that every nurse for her own sake should not let the opportunity pass which is still open to her to come forward without further delay, show herself to be one of those who are grateful to Mr. Stanley, and a believer in the College and all it can accomplish to improve the education, status, protection, and remuneration of British nurses. Every British nurse should make up her mind that she will not, at any rate, wait to come in afterwards as one of the laggards. We have had many unpleasant things said in the House of Commons and elsewhere about shirkers, and the nation expects British nurses to come forward and to avail themselves of the opportunity the College of Nursing offers to them to help themselves.

### The Nation's Fund for Nurses.

There is one other point. The gratitude, or, rather, the feeling of indebtedness, to the nursing profession throughout all classes seeks some means of expression in a practical form. That form has been suggested by the establishment of the Nation's Fund for Nurses, which will have a two-fold object—one, to provide a sum adequate to establish a great Benevolent Fund equal to all the needs of all workers among the sick, and, next, to supply the capital sum which will be necessary to erect a fine block of buildings, complete in every respect, to accommodate a Royal College of British Nursing, so that it may justly claim to be one of the great educational establishments and halls of assembly which the world possesses. Against this just claim to provide adequately for the nursing profession the same small Divide party has tried to throw dirt so as to delay its fulfilment. They declare that nurses will not have charity. Well, it is old days to tell us, who are connected with the management and the early days of the Pension Fund, that nurses will not have charity. We had in the early days of this Fund a great sum of money —over £60,000—which was given by people interested in nurses in order that there might be a reserve fund, and that the nurses might know that they were being thought of and cared for. But the nurses themselves, or many of them, objected to have gifts of that kind presented to them in money at all. The result of this was that we discontinued the raising of further money for that donation fund, and, as the accounts show, it has gradually assumed so small a sum as to be inconsiderable.

### Endowments for Education.

But, on the other hand, when you have a great enterprise like the College, which is in exactly the same position as one of our big universities in its early days, you must have a building, and as our public schools, universities, and other foundations prove, such buildings are in part eleemosynary; that is to say, they have been erected and some of them endowed by people who are interested in education or the particular objects of the foundation. That is all that the nurses can accept or will be offered—*i.e.* the money to erect the College buildings, to complete it, and possibly money to create a Benevolent Fund adequate to the needs of working British nurses who from no fault of their own may fall out by the way and be incapacitated for further work. We have connected with this Fund the Junius S. Morgan Benevolent Fund, and I could only wish that every policyholder in this Fund could sometimes attend or be present at the committee meetings of that Fund, or could have brought to her mind the cases dealt with—the actual work is done steadily, continuously, day by day—for by its efforts we can claim that every deserving nurse who is overtaken by misfortune, or who has to suffer the claims due to the responsibility of friends or relatives who are ill, can through this Fund secure a friend who anonymously and quietly tides her over her difficulties. That is amongst the best work a Benevolent Fund can do. I hope and believe that when the proposed Benevolent Fund for all disabled nurses progresses, means will be taken by which the methods pursued by the Junius S. Morgan Benevolent Fund will be followed in connection with it, for I know no basis better to secure that nurses and the whole nursing profession will obtain by its means the largest possible benefit. I am afraid I have taken up a little of your time, but I wanted to-day to have a talk to you as Pension Fund nurses, and to ask you to look at the position of your profession, to consider, each of you, "What can I do to help my profession? Where do I stand? What step am I going to take to help?" You have many opportunities in taking that step. To register with the College of Nursing is one. I now have great pleasure in seconding the adoption of the report. (Cheers.)

